# Retrospective observational study of chloral hydrate use in mechanically-ventilated pediatric intensive care unit (PICU) patients 2012–2017

**DOI:** 10.3389/fphar.2023.1111528

**Published:** 2023-05-04

**Authors:** Nicholas A. Ettinger, Amy Kiskaddon, Lindsay McNeely, Justin Craycraft, Amber Rogers, Barbara-Jo Achuff, Danielle Guffey, Matthew Musick

**Affiliations:** ^1^ Division of Pediatric Critical Care, Department of Pediatrics, Texas Children’s Hospital, Baylor College of Medicine, Houston, TX, United States; ^2^ Department of Pharmacy, All Children’s Hospital, Johns Hopkins University School of Medicine, St. Petersburg, FL, United States; ^3^ Division of Pediatric Critical Care, Department of Pediatrics, Seattle Children’s Hospital, Seattle, WA, United States; ^4^ Tableau Software, Austin, TX, United States; ^5^ Department of Pediatrics, Texas Children’s Hospital, Baylor College of Medicine, Houston, TX, United States; ^6^ Institute for Clinical and Translational Research, Baylor College of Medicine, Houston, TX, United States

**Keywords:** chloral hydrate (CH), critical care (ICU), mechanical ventilation, pediatric ICU, sedatives and hypnotics

## Abstract

**Introduction:** Chloral hydrate (CH) has long been utilized as a pediatric procedural sedation agent. However, very little is published describing CH use in a pediatric intensive care unit (PICU) setting. The aim of this retrospective observational cohort study was to investigate and describe the use of CH in mechanically-ventilated, critically ill children at a large pediatric tertiary referral hospital.

**Methods:** Data were extracted from the hospital electronic medical record and a locally maintained registry of all children admitted to the PICU between 2012 and 2017. Patients admitted to the cardiovascular ICU were not included in this review. The clinical and pharmacy data for 3806 consecutive PICU admissions of mechanically-ventilated, critically ill children were examined.

**Results:** 283 admissions received CH during their first ICU stay. CH-exposed children were younger (16 months vs. 35 months, *p* < 0.001), the median total dose of CH (indexed to duration of ventilation) was 11 mg/kg/day, the median time to first CH dose was 3 days and more CH doses were administered at night (1112 vs. 958, *p* < 0.001). We constructed a propensity score to adjust for the differences in patients with and without CH exposure using logistic regression including variables of age, sex, diagnosis, and PRISM3 score. After adjustment, the median length of mechanical ventilation was 5 days longer in the CH-exposed group (95% Confidence Interval [CI] 4–6) compared to unexposed CH patients. Similarly, the median length of ICU duration was 9.4 days longer (95% CI 7.1–11.6) and median length of hospital admission duration was 13.2 days longer (95% CI 7.8–18.6) in CH-exposed patients compared to CH-non-exposed. After adjustment, CH-exposed patients had a 9% higher median exposure to HFOV (95% CI 3.9–14.6), but did not have higher median exposures to new tracheostomy (95% CI −0.4–2.2) or ECMO (95% CI −0.2–5.0).

**Discussion:** As part of an extended sedation regimen in mechanically-ventilated and critically ill children, CH is associated with somewhat higher complexity of illness and longer ICU durations.

## 1 Introduction

Chloral hydrate (CH), one of only a few enteral sedating agents, has been used in a variety of inpatient and outpatient clinical settings as a pediatric procedural sedation agent (Heistein et al., 2006; Hill et al., 2016; Reynolds et al., 2016). In 2012, the only remaining commercially available liquid CH product was discontinued in the United States (ISMP. Chloral Hydrate, 2016; Grissinger, 2019). However, some states/institutions still compound an oral suspension for procedural sedation uses from United States Pharmacopeia-grade crystals (Hill et al., 2016). Historically, CH has had mixed popularity and has been associated with unwanted side effects including excessive sedation resulting in respiratory depression, as well as hemodynamic compromise (ISMP. Chloral Hydrate, 2016; Grissinger, 2019). While these effects may be to an extent dose-related, they have reduced its popularity as a sedation agent. There are very limited data describing the use of CH in an intensive care unit setting with mechanically-ventilated, critically ill children (Parkinson et al., 1997; Jenkins et al., 2007; Martinbiancho et al., 2009; Cruise et al., 2012; Staveski et al., 2014; Garcia Guerra et al., 2016; Joffe et al., 2017). During this report’s study period, we prescribed CH in our pediatric intensive care unit (PICU), typically at 25 mg/kg/dose as often as every 6 h as needed, as an adjunct enteral sedation agent for patients already receiving intravenous agents as part of our routine ICU sedation protocol. Given the paucity of PICU-specific data, this retrospective observational cohort analysis serves to describe and characterize the usage of CH from 2012 to 2017 in our mechanically-ventilated, critically ill PICU population and to examine clinical outcomes of CH-exposed children compared to CH-unexposed children.

## 2 Materials and methods

Clinical Data: This is a retrospective observational cohort study of intubated admissions exposed to CH in the then 35-bed PICU at our hospital between 2012 and 2017 (CH ceased to be available in our PICU starting in early 2018). These studies involving human participants were reviewed and approved by the Baylor College of Medicine Institutional Review Board (H-38700). As this was a retrospective analysis of care that had already been delivered, written informed consent was not required to participate in this study in accordance with national legislation and institutional requirements. Virtual Pediatric Systems, LLC (VPS) is a voluntary multicenter national PICU registry database that facilitates comparative pediatric critical care research, quality improvement efforts and benchmarking both within and among institutions (Wetzel et al., 2011). De-identified data are abstracted locally in real-time by trained individuals with critical care experience, undergo a quality assurance process to minimize missing or incorrect data and then are submitted to the national registry. We retrospectively queried our internal VPS database to extract clinical data for all mechanically-ventilated patients from 2012 to 2017. During the period of review, there were 11,492 PICU admissions of which 3,806 were endotracheally intubated and mechanically ventilated during their first ICU admission. Of these 3,806 mechanically ventilated children, 283 were exposed to CH as part of their sedation medications. Exclusion criteria included presence of a tracheostomy at admission to the PICU or patients in the cardiac or neonatal intensive care unit. Data collected included demographic data, pediatric risk of mortality (PRISM-3) scores (Pollack et al., 1996), hospital and ICU admission/discharge date-times, duration of mechanical ventilation as well as other clinical outcomes including mortality. Age stratification for analysis was as follows: age <30 days at ICU admission—neonate; age ≥30 days and <2 years—infant; age ≥2 years and <12 years—pediatric; age ≥12 years—adolescent. Gestational age was not examined in this study, although it is the typical practice of the institution that the majority of children presenting critically ill to the hospital from outside, regardless of age, are admitted to the PICU as opposed to the neonatal intensive care unit. VPS reports primary and secondary diagnoses for patients in the registry. To facilitate comparisons, diagnoses were grouped into larger clinical categories (Pulmonary, Cardiovascular, Sepsis-Shock-Infectious, Neurologic, etc.). At the time of the study, the default PICU intravenous sedation protocol for mechanically-ventilated, critically ill children included fentanyl and midazolam (continuous infusion and bolus doses) as the two initial drugs in the protocol. Dexmedetomidine (continuous infusion) was the typical third line sedation agent. Although available during this time period, CH was not officially part of the standard PICU sedation protocol and was used as an adjunct option for patients who remained inadequately sedated or who were deemed unable (for clinical reasons) to follow the escalation protocol for standard agents and who were able to tolerate enteral medications.

Medication Data: We extracted from our electronic medicine administration record all sedation or paralytic medication data for all of the intubated and mechanically ventilated admissions, including information regarding continuous vs. bolus administration, continuous infusion rates/rate-changes and dosing weights. The admissions were separated into “CH-exposed” and “CH-unexposed” subgroups. All routes of administration of medication were recorded. A Python script was employed to match data from the VPS registry to the medication data from the extract as well as to calculate total dose amounts for continuous infusion medicines. We analyzed and matched the data based on unique admissions as opposed to unique patient medical record numbers. Duration of mechanical ventilation was defined as the total time from time of intubation to time of extubation, expressed in days. ICU duration was the difference of the admission date-time subtracted from the physical-transfer-out-of-the-PICU date-time. To calculate total CH, total opioid and total benzodiazepine exposure for each patient, the total mg dose of each class of medications [opioids converted to morphine equivalents; benzodiazepines converted to midazolam equivalents using standard conversion algorithms (Curley et al., 2015)] was normalized to each patient’s duration of mechanical ventilation [mg of drug/kg weight patient/duration of mechanical ventilation (days) = mg/kg/day]. For each CH-exposed patient, “Time to First Chloral Hydrate Dose” was the date-time for the first CH dose subtracted from the date-time of when the patient was first intubated, expressed in days.

Statistical analysis: For patients with more than one ICU admission or more than one intubation, analysis was limited to the first admission or intubation period (91% of the patients had one admission; 9% of the patients had between two and eight admissions). Demographic variables are summarized as number and percent of total or median with 25th–75th interquartile ranges (IQR). Summary statistics were stratified by choral hydrate exposure and compared using two sample Wilcoxon rank sum test, Fisher’s exact test, or Chi-square test. Additionally, to attempt to account for any bias, a propensity score was constructed to adjust for the differences in patients with and without CH exposure, using logistic regression including variables of age, sex, diagnosis category, and PRISM3 score. The propensity score was used to match patients by CH exposure 1:2 using the nearest neighbor estimator with the caliper width set to 0.03 of the standard deviation of the logit of the propensity score. Covariate balance before and after matching was examined using standardized differences, with values 0.15 considered as evidence of meaningful differences. The average treatment effect/difference is computed and presented with 95% confidence intervals (CI). Data were compiled with the business informatics software, Tableau™ and statistical analysis performed using Stata v15.

## 3 Results

Cohort clinical data: Clinical data for all mechanically ventilated, critically ill CH-exposed and CH-unexposed children from 2012 to 2017 are presented in Table 1. There were 283 total admissions exposed to CH (7.4%) and 3,523 total admissions not exposed to CH. CH-exposed patients were younger and had a higher proportion of infants and a lower proportion of adolescents compared to CH-unexposed patients (Table 1). 51.9% of CH-exposed patients and 57.5% of CH-unexposed patients were male. VPS clinical diagnosis groupings did differ statistically between CH-exposed and CH-unexposed patients (Table 1), with proportionally more CH-exposed patients in the VPS clinical groupings of “Sepsis-Shock-Infectious” and “Pulmonary,” but not the “Neurologic” grouping (the three largest VPS clinical groupings overall). CH-exposed patients did not have higher PRISM-3 scores at admission.

**TABLE 1:** Cohort clinical data.

Characteristic	CH-exposed (*n* = 283)		CH-unexposed (*n* = 3,523)		*p*-value^a^
Age at ICU admission (months)^b^	16 (8, 35)		35 (8, 125)		*p* < 0.001
Age Groupings					
Neonate (< 30d)^c^	3 (1.1%)		99 (2.8%)		*p* < 0.001
Infant	183 (64.7%)		1,457 (41.4%)		
Pediatric	86 (30.4%)		1,214 (34.5%)		
Adolescent	11 (3.9%)		753 (21.4%)		
			
Gender^c^					
Male	147 (51.9%)		2,026 (57.5%)		*p* = 0.071
Female	136 (48.1%)		1,497 (42.5%)		
			
VPS Primary Diagnosis Groupings^c^					
Airway	25	(8.8%)	150	(4.3%)	*p* < 0.001
Cardiovascular	6	(2.1%)	113	(3.2%)	
Gastrointestinal/Liver/Endocrine	14	(4.9%)	220	(6.2%)	
Hematology-Oncology/Inflammatory	4	(1.4%)	157	(4.5%)	
Neurologic	23	(8.1%)	1,018	(28.9%)	
Poisoning/Ingestion	0	(0.0%)	82	(2.3%)	
Pulmonary	85	(30.0%)	679	(19.3%)	
Renal	3	(1.1%)	54	(1.5%)	
Sepsis/shock/infectious	120	(42.4%)	905	(25.7%)	
Trauma/accident	1	(0.4%)	108	(3.1%)	
Other	2	(0.7%)	37	(1.1%)	
PRISM-3 Score	6 (3, 10)		5 (3, 10)		*p* = 0.656

^a^ Statistical testing: Summary statistics were stratified by choral hydrate exposure and compared using two sample Wilcoxon rank sum test for median comparisons or exact testing for categorical variables when possible, otherwise chi-square test.

^b^ Median (IQR).

^c^ Number (Percent).

Cohort Outcome Data: Table 2 shows outcomes data in the CH-exposed admissions vs. CH-unexposed admissions. After propensity score matching on patient age, sex, diagnosis category, and PRISM-3 score (Table 2, see Methods for statistical details), patients exposed to CH had a median of 5 days longer mechanical ventilation (95% CI 4–6) compared to unexposed CH patients. Similarly, after propensity-score matching, patients exposed to CH had a median of 9.4 days longer ICU duration (95% CI 7.1–11.6) and a median of 13.2 days longer overall hospital duration (95% CI 7.8–18.6). After propensity matching, patients exposed to CH had a median 9% higher exposure to HFOV (9.3%, 95% CI 3.9–14.6), but did not have higher median exposures to new tracheostomy (95% CI −0.4 to 2.2) or ECMO (95% CI −0.2 to 5.0). There was not a statistically significant difference in mortality rates with CH-exposure (95% CI −2.6 to 5.7).

**TABLE 2:** Cohort outcome data.

Characteristic	CH-exposed (*n* = 283)	CH-unexposed (*n* = 3,523)	Propensity score matched difference (95% CI) *p*-value^a^
MV duration (days)^b^	6.7 (4.1,13.2)	2.3 (0.7,5.2)	5.0 (4.0, 6.0) <0.001
ICU duration (days)	11 (6.2,22.8)	4.6 (2.1,8.8)	9.4 (7.1, 11.6) <0.001
Hospital duration (days)	19.9 (11.1,39.2)	11 (5.4,22.4)	13.2 (7.8, 18.6) <0.001
Mortality^c^	30 (10.6%)	293 (8.3%)	1.5% (−2.6, 5.7) 0.462
Exposed to.?			
High frequency oscillation ventilation	42 (14.8%)	178 (5.1%)	9.3% (3.9, 14.6) 0.001
Tracheostomy	11 (3.9%)	48 (1.4%)	0.9% (−0.4, 2.2) 0.186
ECMO	13 (4.6%)	43 (1.2%)	2.4% (−0.2, 5.0) 0.070

^a^ Statistical testing: Summary statistics of the cohort outcomes were stratified by choral hydrate exposure and compared using propensity score matched median treatment difference.

^b^ Median (IQR).

^c^ Number (Percent).

**FIGURE 1:**
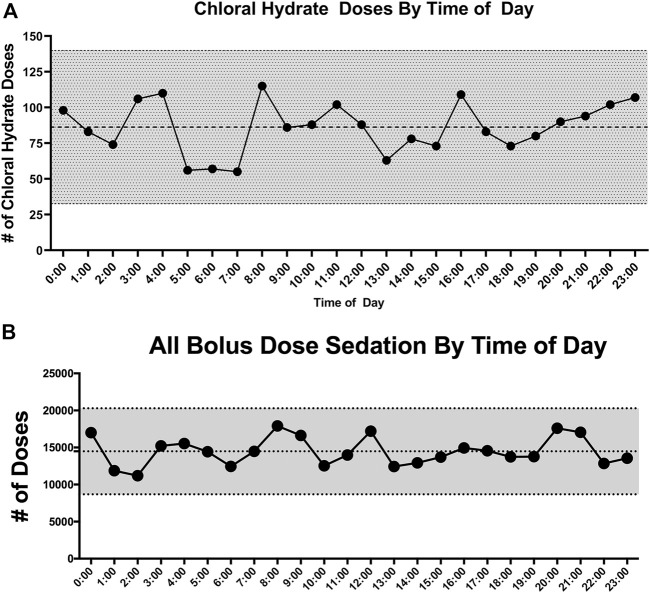
**(A)** Control chart of choral hydrate doses by hour of the day. The number of doses for each hour of the day is plotted vs. hour of the day. The mean number of doses is represented by the middle dotted line. The outer dotted lines and gray shading represent ± 3 standard deviations from the mean. **(B)** Control chart of all bolus dose sedation by hour of the day. The total number of bolus sedation doses for each hour of the day is plotted vs. hour of the day. The mean number of doses is represented by the middle dotted line. The outer dotted lines and gray shading represent ± 3 standard deviations from the mean.

**FIGURE 2:**
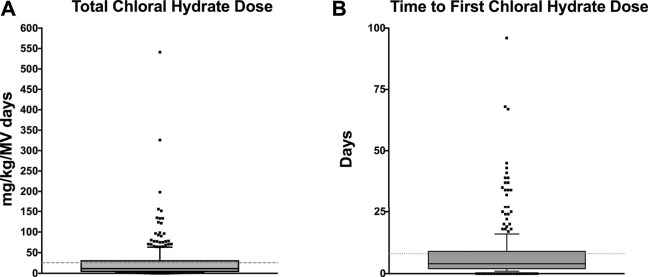
**(A)** Box-and-Whisker plot of the total amount of chloral hydrate per patient (mg/kg/MV days). The total mg/kg of CH per patient is indexed to each patient’s length of mechanical ventilation. The grey shaded box represents the 25th–75th IQR and the dark middle line represents the median. The dotted line represents the mean. Individual values outside the 10%–90% whiskers shown as individual points. **(B)** Box-and-Whisker plot of time-to-first-chloral-hydrate dose (days). The day of first CH dose for each patient is shown as a box-and-whisker plot. The grey shaded box represents the 25th–75th IQR and the dark middle line represents the median. The dotted line represents the mean. Individual values outside the 10%–90% whiskers shown as individual points.

Medication Administration Data: Table 3 lists medication data. As CH was administered as an adjunct agent, we wanted to examine the chronological pattern of CH dosing compared to other sedation medications. CH doses were more often administered at night [7pm–7am] (*p* < 0.001) which was consistent with the overall pattern of bolus dose sedation administration [neuro-muscular blockade medications not included] (*p* < 0.001). When we examined the chronological pattern of either CH dosing (Figure 1A) or all bolus sedation dosing (Figure 1B) with a quality-improvement-style control chart over the day by hour-of-the-day, we did not observe any statistically significant dose administration outliers. After propensity-score matching, CH-exposed patients received on average two more classes of sedative medications (95% CI 2.2–2.7). To compare total exposures to sedation medications, we calculated the total daily amount per patient of CH, opioid and benzodiazepine administered indexed to each patient’s length of mechanical ventilation (mg/kg/day). As shown in Table 3, after propensity score matching, CH-exposed patients received a median of 2.6 mg/kg/day more of total midazolam equivalents (95% CI 1.6–3.6) and 0.7 mg/kg/day more of total morphine equivalents (95% CI 0.5–1.0) consistent with this subgroup’s longer duration of mechanical ventilation and longer ICU durations. As shown in the box-whisker plot in Figure 2A, the distribution of total CH dosing per patient was skewed with a median of 11 mg/kg/day and a mean of 26 mg/kg/day. To examine the variation of when providers were adding CH to each patient’s sedation regimen, we calculated the time-to-first-chloral-dose to see if there was a consistent pattern. As shown in the box-whisker plot in Figure 2B, the median time (after intubation) to receive CH was 3 days and the mean was 7 days. Among the different VPS clinical groupings, there were no statistical differences in median time-to-first-CH dose (data not shown).

**TABLE 3:** Medication administration data.

Time of day administered	Day 7a.m.–7p.m.	Night 7p.m.–7a.m.	*p*-value^a^
	N (%)	N (%)	
# of chloral hydrate doses^b^	958 (46%)	1,112 (54%)	*p* < 0.001
# of all bolus sedation doses (excluding neuromuscular blockade)^b^ ^,^ ^c^	160,378 (46%)	186,794 (54%)	*p* < 0.001
Number of sedative classes received?^c^	CH-exposed (*n* = 283)	CH-Unexposed (*n* = 3,523)	Propensity score matched difference (95% CI) *p*-value^d^
Mean (SD)	5.7 (1.2)	3.3 (1.2)	2.4 (2.2, 2.7) <0.001
Daily medication totals^e^			
Daily choral hydrate dose (mg/kg/day)	11.4 (4.6, 30.2)	0 (0, 0)	11.8 (10.2, 13.5) <0.001
Daily midazolam equivalents (mg/kg/day)	4.4 (2.5, 7.3)	1.7 (0.3, 4.5)	2.6 (1.6, 3.6) <0.001
Daily morphine equivalents (mg/kg/day)	1.1 (0.6, 3.0)	0.4 (0.2, 0.9)	0.7 (0.5, 1.0) <0.001

^a^ Statistical testing: Mean comparisons performed with t-test. Summary statistics stratified by day and night shift and compared using two sample Wilcoxon rank sum test for median comparisons or exact testing for categorical variables when possible, otherwise chi-square test.

^b^ Data presented as Number (Percent), all 3,806 patients.

^c^ Sedation medication classes: Opioid (fentanyl, morphine, hydromorphone, methadone), Benzodiazepine (midazolam, lorazepam), Alpha-agonist (Clonidine, Dexmedetomidine), Propofol, Ketamine, Chloral Hydrate, Barbiturate (pentobarbital, phenobarbital), Neuromuscular Blocker (any).

^d^ Statistical testing: Summary statistics for medication data stratified by chloral hydrate exposure and compared propensity score matched average or median treatment difference.

^e^ Data presented as Median (IQR).

## 4 Discussion

The purpose of this retrospective observational cohort analysis was to describe and characterize the usage of CH in mechanically-ventilated, critically ill PICU patients and to examine clinical outcomes of CH-exposed children compared to CH-unexposed children. Our data show that, as part of an extended sedation regimen and its use as an adjunct sedation agent, CH exposure is not surprisingly associated with higher complexity of illness and longer ICU durations.

Historically, CH has been used to sedate pediatric patients in a variety of specific procedural sedation settings (echocardiography, hearing tests, MRI, etc.) (Hill et al., 2016; Reynolds et al., 2016). CH is an enteral sedative-hypnotic which, after administration, is rapidly reduced to the active compound of trichloroethanol. Trichloroethanol exerts a barbiturate-like effect via the GABA_A_ receptor channels (Mihic et al., 2017). Onset of action is typically 15–30 min and sedation lasts for 1–2 h (Krauss and Green, 2006). Importantly, CH has no analgesic properties.

There are very few reports describing the use of CH as a sedation agent for critically ill children in a pediatric ICU setting. Therefore, we sought to describe and characterize the usage of CH in our PICU. At the time of the study, CH was not used as first-line therapy for sedation for mechanically-ventilated, critically ill children, rather it was used as an adjunct. We found that CH was used in younger children (Table 1) and, consistent with its use as an adjunct, CH-exposure tracked with markers of overall higher severity of illness—longer hospital, ICU and mechanical ventilation durations (Table 2) and exposure to higher total numbers of sedation medication classes compared to non-exposed patients (Table 3). CH-exposure was also associated with receiving more bolus sedation doses at night, although there was not a specific hourly-pattern to either CH-dosing or bolus-sedation dosing (Figure 1). CH-exposure was also associated with exposure to more complex PICU therapies such as high-frequency ventilation (Table 2), suggesting that CH is used in sicker patients who, as a result of their underlying disease state, require more complex ICU-level therapies and have longer lengths of stay. Reassuringly, CH-exposure was not associated with a higher percentage of mortality (Table 2). Total CH-dosing per patient per admission was significantly skewed with a median of 11 and a mean of 26 mg/kg/day. However, there were a striking number of patients who received significantly larger doses of CH with total indexed doses ≥150 mg/kg/day (Figure 2A). Lastly, when we looked at time-to-first CH dose (Figure 2B) among the larger clinical VPS groupings (Table 1), we did not observe any differences (data not shown) suggesting that one clinical subgroup was not being sedated categorically differently to other subgroups.

Non-procedural, PICU-sedation use of CH has only been studied sporadically. In 1997, Parkinson et al. (1997) performed a small randomized trial in mechanically ventilated children comparing standard midazolam sedation versus a combination of CH and promethazine and found that the combination regimen potentially performed better than just midazolam. Jenkins et al. (2007) employed a prospective observational study via survey to examine sedation practices in PICUs across the United Kingdom. They demonstrated that there was considerable heterogeneity among unit practices. Of 360 children examined, 93 had exposure to CH and it was the most common enteral sedation medication employed across the study. Garcia Guerra et al. (2016) surveyed Canadian pediatric critical care physicians and found that CH was one of the most commonly used adjunct sedation agents. Martinbiancho et al. (2009) examined the use of CH for prolonged sedation in PICU patients in Brazil. They examined all CH-exposed patients in their PICU (mechanically-ventilated and non-ventilated patients) and their primary outcome was adverse drug events, with the most common adverse event being oxygen desaturation. Only 58.6% of their PICU population were mechanically-ventilated with a median intubation duration of 2 days (we restricted our analysis to intubated, mechanically ventilated patients; median intubation duration for our PICU is 2.8 days, unpublished data). Median total dose per patient was 130 mg/kg/day, a much higher exposure than for most of the patients in our study (median 11 mg/kg/day; mean 26 mg/kg/day), where the drug is used as an adjunct, as opposed to a primary modality. Cruise et al. (2012) examined CH use in a neonatal ICU context and found that more doses were given at night and above their institution’s dosing recommendations, raising concerns of how it was being used by clinicians. Similarly, Staveski et al. (2014) found that CH was used more often at nighttime in a cardiac ICU. We also found a significant difference in bolus sedation dosing between day shift and night shift with more bolus doses of all sedation agents given at night time (Table 3). Diurnal variation in sedation dosing has been studied in adults, demonstrating higher doses of opioids and benzodiazepines at night potentially delaying spontaneous breathing trials and extubation (Mehta et al., 2016) and with limited data in children, who did not receive higher doses at night in a single center study (Loberger et al., 2021). Joffe et al. (2017) performed a pilot feasibility study providing continuous enteral CH solution (5 mg/kg/hr). They demonstrated that all 21 patients tolerated the infusion (primary outcome) and that the numbers of as-needed doses decreased in patients receiving the CH infusion, compared to historical controls. These patients also received significantly higher CH doses than the patients in our study with total daily doses ranging from 116 to 160 mg/kg/day. Our patients were exposed to a mean and median of 26 and 11 mg/kg/day, respectively (Figure 2A).

There are several limitations to this study. We were unable to track any information on the incidence or prevalence of important modifiers (like pediatric ICU delirium) as formal sedation scoring, iatrogenic withdrawal scoring and formal delirium screening were not yet performed in our ICU during the period when these data were collected, limiting our ability to address more complex questions of “improved sedation” or the etiology of why more bolus doses were administered at night. Additional limitations include that it is a single center, retrospective analysis restricting its generalizability and its ability to draw causal inference. This study was not powered for analysis of risks of mortality with CH-use. We deliberately restricted our analysis to mechanically ventilated patients to focus the analysis and to obviate the need to worry about the most commonly reported procedural-sedation-related side effect of CH, respiratory depression. We also did not have any concomitant vasoactive-use, hemodynamic or oxygen-saturation data to be able to make a more comprehensive assessment of the hemodynamic effects of CH in our mechanically ventilated population.

## 5 Conclusion

CH is understudied in PICU populations. Although used extensively outside the United States, commercial formulations of CH are not currently available in the United States. Our results examining the use of CH in mechanically-ventilated, critically ill children is notable for its long duration, large data set, and lack of significant “danger” signals. We found that, consistent with its use as an adjunct agent in an extended sedation regimen, CH was associated with higher complexity of illness and longer ICU durations. In December 2016, the FDA published a Drug Safety Communication warning about the possible long-term developmental effects on young children from typical general anesthesia and PICU sedation medications (benzodiazepines, propofol, ketamine, barbiturates) (FDA. Fda, 2356). Notably, CH was not on this list although that does not mean its safety for prolonged sedation has been established. Given that all currently available sedation medications have potential negative side effects that can limit use (including potentially CH) combined with the modern “benzodiazepine-sparing” approach to pediatric sedation in the PICU, we suggest that CH deserves further controlled comparative investigation as a primary or adjunct sedation agent to study measures of its safety (e.g., cardiorespiratory effects, unplanned extubation incidence, incidence of withdrawal and delirium) and efficacy (e.g., sedation scores, delirium scores, duration of mechanical ventilation) in mechanically ventilated, critically ill children.

## Data Availability

The raw data supporting the conclusion of this article will be made available by the authors, by appropriate request, without undue reservation.
